# Supplementary material for: Porous SiC and SiC/Cf Ceramic Microspheres Derived from Polyhydromethylsiloxane by Carbothermal Reduction
Source: Materials (Basel). 2021 Dec 23;15(1):81. doi: 10.3390/ma15010081 (PMC8745781; doi:10.3390/ma15010081)
Supplement: Supplementary file 1 [file materials-15-00081-s001.zip › materials-1451522-supplementary.pdf]

materials

MDPI

Supplementary Materials

Porous SiC and SiC/C_f Ceramic Microspheres Derived from Polyhydromethylsiloxane by Carbothermal Reduction

Mizerska Urszula [1,*], Fortuniak Witold [1,*], Chojnowski Julian [1], Rubinsztajn Slawomir [1], Zakrzewska Joanna [1], Bak-Sypien Irena [1] and Nyczyk-Malinowska Anna [2]

[1] Center of Molecular and Macromolecular Studies, Polish Academy of Sciences, ul. Sienkiewicza 112, 90-363 Lodz, Poland; wfortuni@cbmm.lodz.pl (F.W.); jchojnow@cbmm.lodz.pl (C.J.); srubin@cbmm.lodz.pl (R.S.); jzakrzew@cbmm.lodz.pl (Z.J.); sypieni@cbmm.lodz.pl (B.-S.I.)

[2] Faculty of Materials Science and Ceramics, AGH-University of Science and Technology, Al. Mickiewicza 30, 30-059 Krakow, Poland; nyczyk@agh.edu.pl

* Correspondence: mizerska@cbmm.lodz.pl

Supporting information

Synthesis of preceramic microspheres

The microspheres were synthesized by a method similar to that described in Ref.1 Below is the description of an example of these syntheses.

The 20 w% Pt Karstedt complex solution containing 4.8 x 10^{-6} mol of Pt was introduced to 48.1 g of PHMS and 38.8g (0.30 mol) of DVB dissolved in 40 mL of dioxane kept at 45 °C. The solution was stirred until the temperature of 50 °C was reached. At that point it was homogenized with 400 mL of distilled water containing 0.4 % of PVA at 45 °C using MPW-120 homogenizer set to 7500 rpm for 90 s. The obtained emulsion was diluted with 3200 mL of degassed water containing 0.4% of PVA and gently stirred at 45 °C for 50 h. The microspheres formed were isolated by centrifugation. Subsequently the particles were washed with water several times and finally freeze-dried. 72.82 g of microspheres was obtained, 84% yield. Spectroscopic characterization of various samples of preceramic microspheres is displayed in Table S1 and SEM micrographs of selected samples are shown in Figure S1 while examples of size distribution are shown in Figure S2.

Table S1. Spectroscopic characterization of the pre-ceramic microspheres obtained by a homogenization at 7500 rpm for 90 s.

Sample	^{29}Si CP/MAS NMR Corrected to HPDec					^{13}C CP/MAS NMR
	$SiOH/SiH_0$ %	$Si\text{-}CC/SH_0$ %	$SiOSi/SiH_0$ %	SiH/SiH_0 %	SiOSi/SiCC	$Si\text{-}CC/SiH_0$ %
PA-1	32	15	23	30	1.53	14
PA-2	39	29	10	22	0.34	38
PA-3	21	41	3	35	0.07	38
PA-4	3	42	1	54	0.02	38
PA-5	3	42	2	53	0.05	41.8
PA-6	0	49	0	51	0	48.6
PB-1	43	14	31	12	2.21	

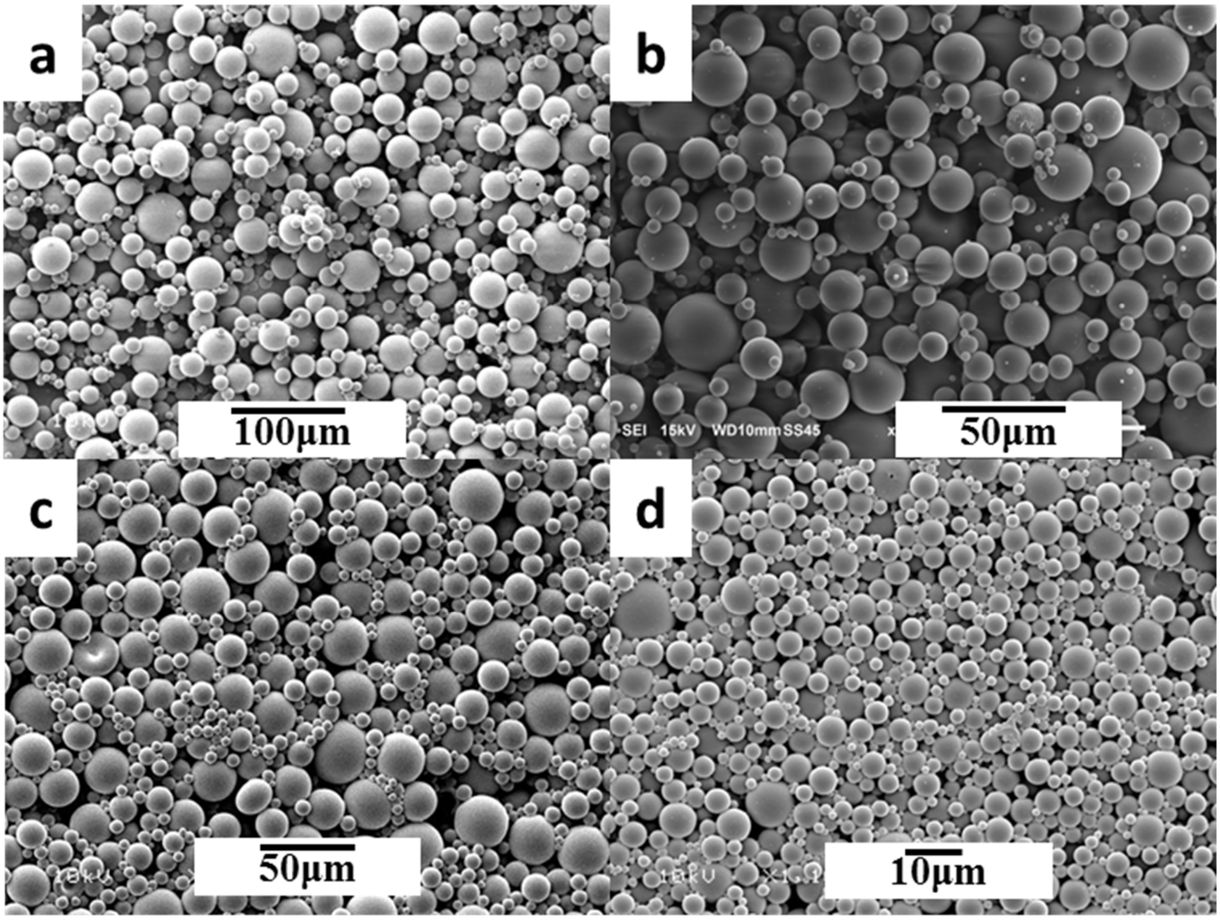

Figure S1. Micrographs of preceramic siloxane microspheres obtained at different DVB/SiH weight ratio: (**a**) PA-1, (**b**) PA-3, (**c**) PB-1, (**d**) PA-6.

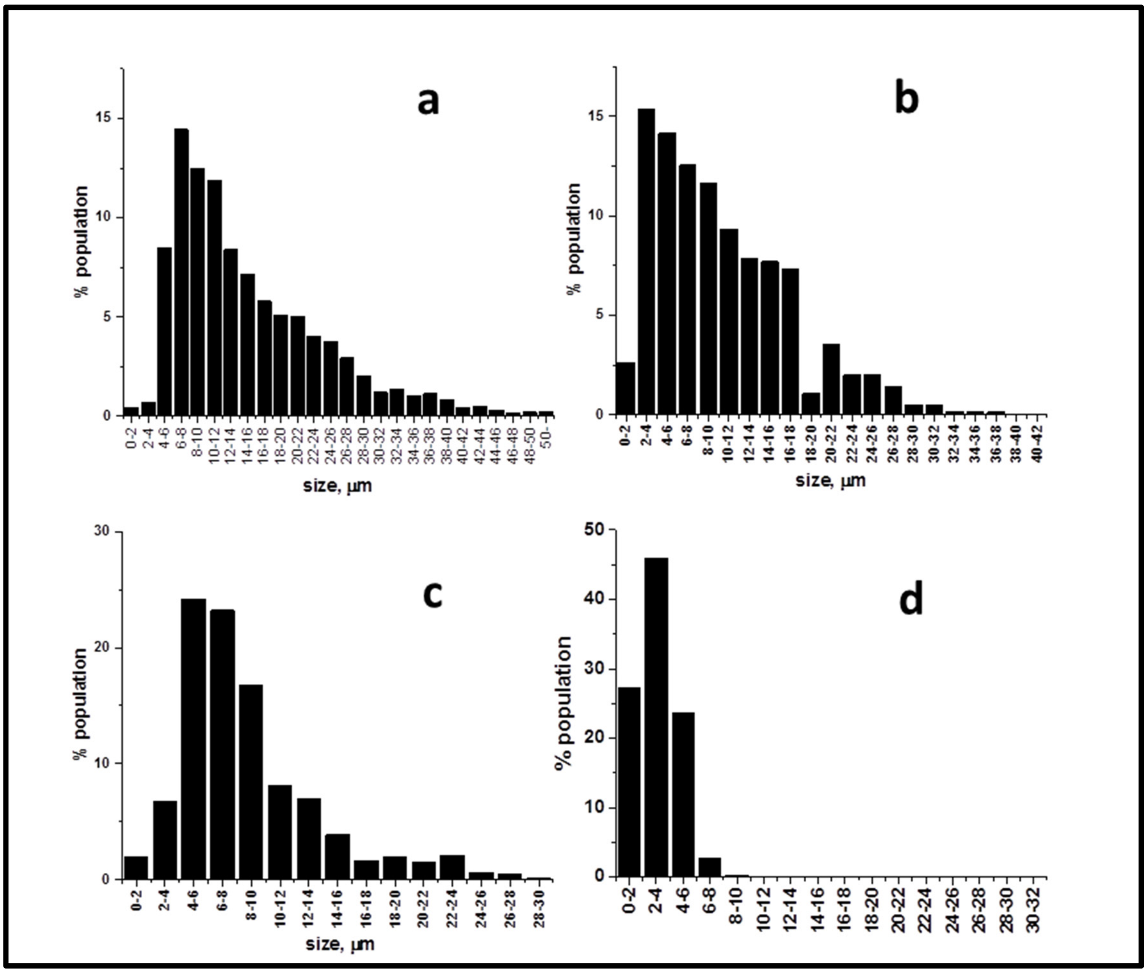

Figure S2. Distribution of sizes of preceramic siloxane microspheres: (**a**) - PA-1, (**b**) - PA-3, (**c**) - PB-1, (**d**) - PA-6.

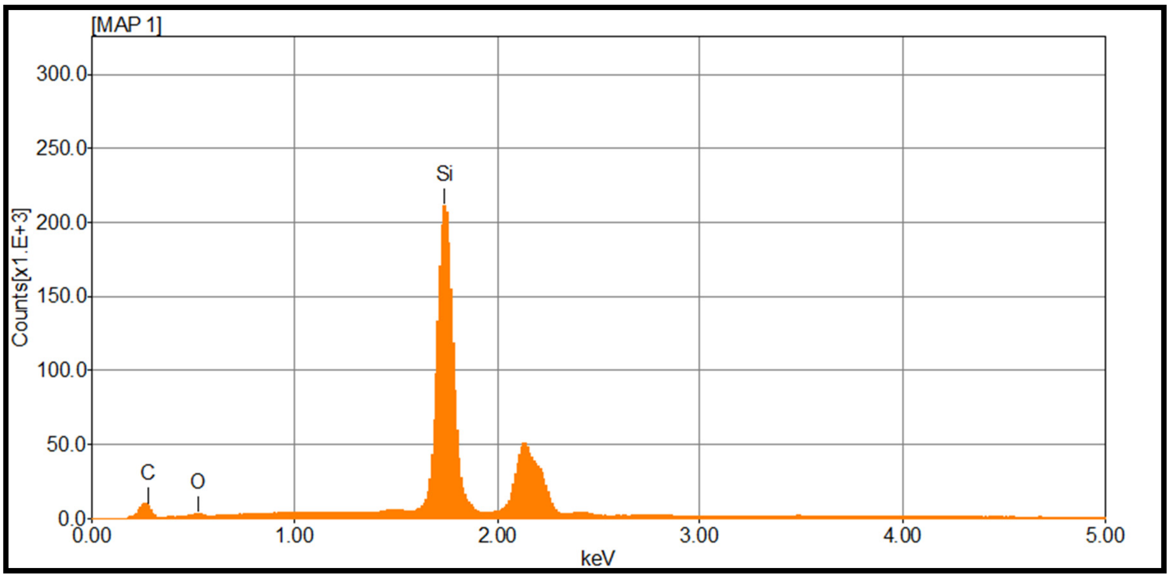

Figure S3. Example of SEM/EDS spectrum of ceramic microspheres: sample A- 6.

Table S2. Elemental composition of the ceramic microspheres determined by SEM/EDS.

Ceramized sample	w% C_{total}	w% O	w% Si	Calculated w% C_f
A-1	28.17	2.98	68.86	0.20
A-2	30.46	2.46	67.08	2.60
A-3	29.49	4.10	66.41	2.60
A-4	37.38	2.91	59.70	12.40
A-5	36.09	1.12	62.53	10.00
A-6	37.80	2.10	60.10	12.90

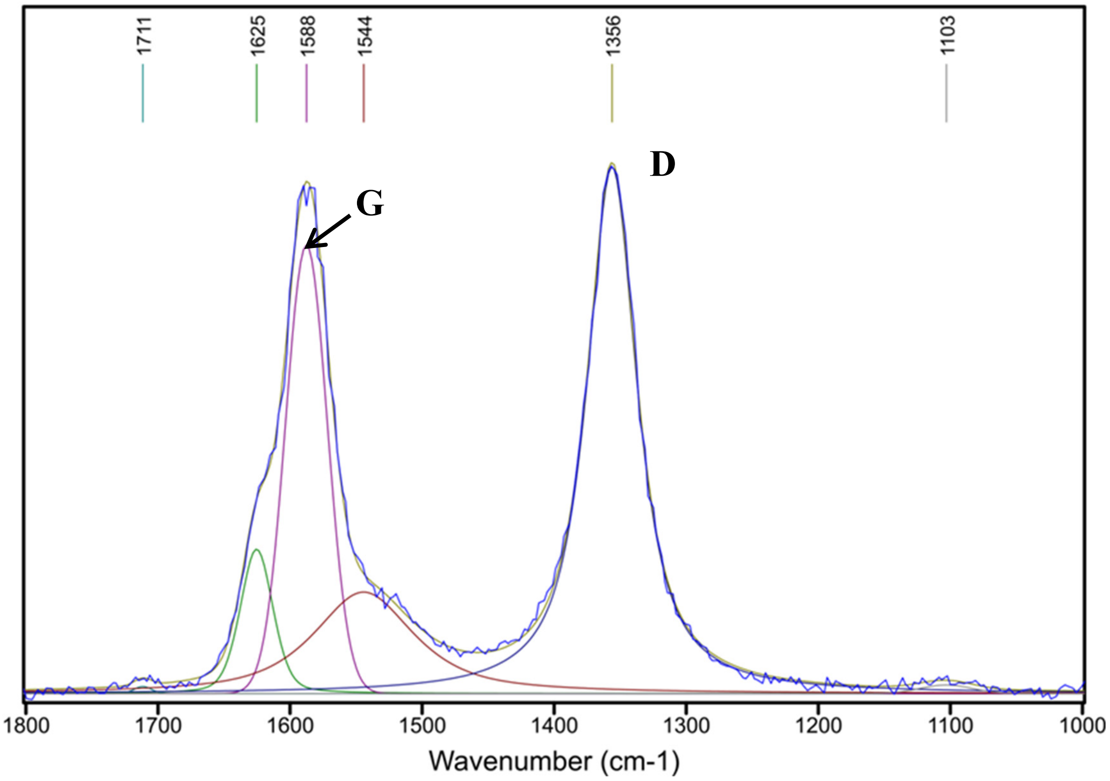

Figure S4. Example of Raman spectrum of the SiC/C_f ceramic microspheres – sample A-5 DVB/HMPS w/w = 0.80, the resolution of the overlapping bands was made using Gaussian-Lorentzial approach.

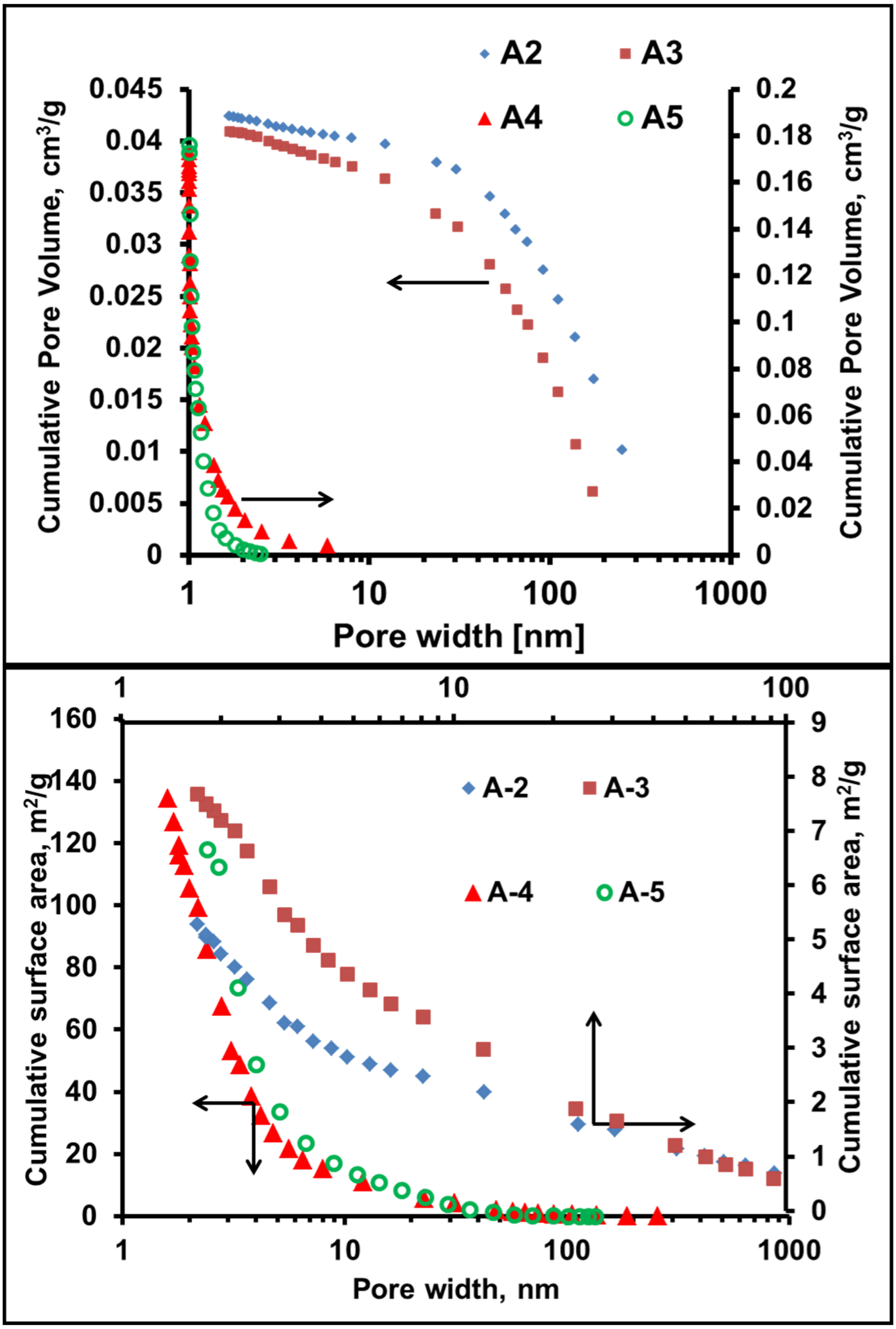

Figure S5. Cumulative plots of the distribution of pore width: (**Top**) by pore volume, (**bottom**) by pore surface.

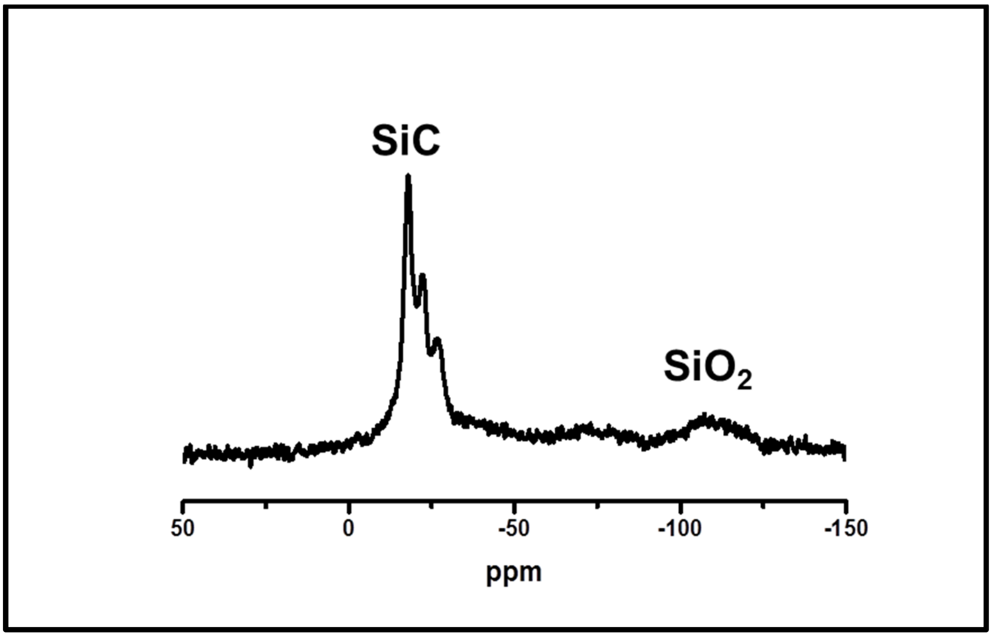

Figure S6. ^{29}Si MAS NMR spectrum of ceramic microspheres A-7 after heating in air at 800 °C for 3 h.

References

1. W.Fortuniak, J.Chojnowski, S.Slomkowski, P.Pospiech, J.Kurjata. Route to hydrophilic, hydrophobic and functionalized cross-linked polysiloxanes microspheres. *Polymer*. **2013**, *54*, 3156-3165. https://doi.org/10.1016/j.polymer.2013.04.017
